# Horizontal Gene Transfer of Antibiotic Resistance from Acinetobacter baylyi to Escherichia coli on Lettuce and Subsequent Antibiotic Resistance Transmission to the Gut Microbiome

**DOI:** 10.1128/mSphere.00329-20

**Published:** 2020-05-27

**Authors:** Marlène Maeusli, Bosul Lee, Sarah Miller, Zeferino Reyna, Peggy Lu, Jun Yan, Amber Ulhaq, Nicholas Skandalis, Brad Spellberg, Brian Luna

**Affiliations:** a Department of Medicine, Keck School of Medicine, University of Southern California, Los Angeles, California, USA; b Department of Molecular Microbiology and Immunology, Keck School of Medicine, University of Southern California, Los Angeles, California, USA; c Los Angeles County + University of Southern California Medical Center, Los Angeles, California, USA; Antimicrobial Development Specialists, LLC

**Keywords:** One Health, agriculture, antibiotic, antimicrobial resistance, gene transfer, host-microbe, microbiome, plant-microbe interactions

## Abstract

Agricultural use of antibiotics is recognized by the U.S. Centers for Disease Control and Prevention as a major contributor to antibiotic-resistant infections. While most One Health attention has been on the potential for antibiotic resistance transmission from livestock and contaminated meat products to people, plant foods are fundamental to the food chain for meat eaters and vegetarians alike. We hypothesized that environmental bacteria that colonize plant foods may serve as platforms for the persistence of antibiotic-resistant bacteria and for horizontal gene transfer of antibiotic-resistant genes. Donor Acinetobacter baylyi and recipient Escherichia coli were cocultured *in vitro*, *in planta* on lettuce, and *in vivo* in BALB/c mice. We showed that nonpathogenic, environmental A. baylyi is capable of transferring plasmids conferring antibiotic resistance to E. coli clinical isolates on lettuce leaf discs. Furthermore, transformant E. coli from the *in planta* assay could then colonize the mouse gut microbiome. The target antibiotic resistance plasmid was identified in mouse feces up to 5 days postinfection. We specifically identified *in vivo* transfer of the plasmid to resident Klebsiella pneumoniae in the mouse gut. Our findings highlight the potential for environmental bacteria exposed to antibiotics to transmit resistance genes to mammalian pathogens during ingestion of leafy greens.

**IMPORTANCE** Previous efforts have correlated antibiotic-fed livestock and meat products with respective antibiotic resistance genes, but virtually no research has been conducted on the transmission of antibiotic resistance from plant foods to the mammalian gut (C. S. Hölzel, J. L. Tetens, and K. Schwaiger, Pathog Dis 15:671–688, 2018, https://doi.org/10.1089/fpd.2018.2501; C. M. Liu et al., mBio 9:e00470-19, 2018, https://doi.org/10.1128/mBio.00470-18; B. Spellberg et al., NAM Perspectives, 2016, https://doi.org/10.31478/201606d; J. O’Neill, Antimicrobials in agriculture and the environment, 2015; Centers for Disease Control and Prevention, Antibiotic resistance threats in the United States, 2019). Here, we sought to determine if horizontal transmission of antibiotic resistance genes can occur between lettuce and the mammalian gut microbiome, using a mouse model. Furthermore, we have created a new model to study horizontal gene transfer on lettuce leaves using an antibiotic-resistant transformant of A. baylyi (Ab^zeoR^).

## Abstract (precis)

Previous efforts have correlated antibiotic-fed livestock and meat products with respective antibiotic resistance genes, but virtually no research has been conducted on the transmission of antibiotic resistance from plant foods to the mammalian gut (C. S. Hölzel, J. L. Tetens, and K. Schwaiger, Pathog Dis 15:671–688, 2018, https://doi.org/10.1089/fpd.2018.2501; C. M. Liu et al., mBio 9:e00470-19, 2018, https://doi.org/10.1128/mBio.00470-18; B. Spellberg et al., NAM Perspectives, 2016, https://doi.org/10.31478/201606d; J. O’Neill, Antimicrobials in agriculture and the environment, 2015; Centers for Disease Control and Prevention, Antibiotic resistance threats in the United States, 2019). Here, we sought to determine if horizontal transmission of antibiotic resistance genes can occur between lettuce and the mammalian gut microbiome, using a mouse model. Furthermore, we have created a new model to study horizontal gene transfer on lettuce leaves using an antibiotic-resistant transformant of A. baylyi (Ab^zeoR^).

## OBSERVATION

The U.S. Centers for Disease Control and Prevention (CDC) has estimated that 20% of antibiotic-resistant infections in the United States are attributable to agricultural use of antibiotics (1–3). The majority of antibacterial agents (including ionophores) purchased in the United States have been for use in agricultural settings (15.4 million kg, or 80% of the U.S. annual total in 2014) (1, 4). The use of such agents causes selective pressure, favoring the survival of antibiotic-resistant bacteria and the spread of resistance-conferring genes among livestock (5).

Most of the scientific focus on antibiotic resistance in agriculture has been on livestock and meat products (6–9). However, the plant phyllosphere (leaf surface) and rhizosphere (root system) serve as habitats for environmental microbiota and as hosts of pathogenic plant and animal bacteria (10). Acinetobacter baylyi, a relative of the clinically important and highly antibiotic-resistant human pathogen Acinetobacter baumannii, is a nonpathogenic lettuce colonizer (10, 11). A. baylyi has recently been shown to transfer DNA to diverse bacteria through the secretion of vesicles loaded with plasmid DNA into the environment (12, 13). Gene transfer is thus carried out by non-conjugation-mediated mechanisms that allow broader transmission of plasmids (12, 14). Acinetobacter species are highly pertinent to the issue of multidrug resistance, making A. baylyi a potential vehicle for mediating transfer of antibiotic resistance genes from agriculture to mammals ingesting plants (15).

Horizontal transfer of genetic information has been well studied for transfer of genes between pathogenic bacteria and within environmental communities as well (16). A thorough review of resistant plasmid characteristics within *Enterobacteriaceae* has recently been published by Partridge et al. (17). However, the transfer of genes from nonpathogenic to pathogenic bacteria deserves more attention (12, 18). We hypothesize that environmental bacteria that colonize produce may serve as platforms for the persistence of antibiotic-resistant bacteria and for the horizontal transfer of antibiotic resistance genes to the mammalian gut microbiome.

## 

### *In vitro* transfer of antibiotic resistance genes.

We tested whether transfer of an antibiotic resistance gene could be observed *in vitro* from the A. baylyi donor strain Ab^zeoR^ to a panel of candidate Escherichia coli clinical isolates that was composed of both extended-spectrum beta-lactamase (ESBL)-producing and non-ESBL-producing isolates (Table 1; also, see Fig. S1 and Table S1 in the supplemental material). *In vitro* cocultures were first performed in liquid broth and then enumerated on doubly selective agar plates. Dual antibiotic resistance emerged, confirming successful transformation of numerous strains (Table 1; also, see Fig. S1A). Some spontaneous mutations were also observed in other recipient strains (56663C and 56307C) in broth cocultures that mimicked the phenotype of dual resistance but were not caused by plasmid transmission (Fig. S1A). There was no statistically significant difference between the frequencies of dual resistance in broth among the ESBL-producing and non-ESBL-producing recipients (Mann-Whitney test, *P* > 0.05).

**TABLE 1:** *In vitro* and *in planta* lettuce coculture with Ab^zeoR^

Condition	E. coli recipient	ESBL^*a*^	Avg frequency of dual resistance	PCR confirmation
*In vitro* + broth	DH5αC	N	1.45E−05	Positive
*In vitro* + broth	JJ2528	Y	1.67E−06	Positive
*In vitro* + broth	267-18-50927	Y	3.53E−07	Positive
*In vitro* + broth	330-18-62584	Y	2.83E−07	Positive
*In vitro* + broth	56428	Y	1.24E−01	Positive
*In vitro* + broth	56307C	N	1.12E−06	Positive
*In vitro* + broth	56459C	N	4.91E−07	Positive
*In vitro* + broth	56303C	N	8.04E−07	Positive
*In vitro* + broth	56663C	N	2.68E−07	Positive
*In vitro* + broth	JJ1886	Y	9.85E−07	Negative
*In vitro* + broth	JJ2555	Y	7.17E−07	Negative
*In vitro* + broth	267-19-46076	Y	8.92E−02	Negative
*In vitro* + broth	267-19-44723	Y	8.04E−07	Negative
*In vitro* + agar	JJ2528	Y	3.54E−05	Positive
*In vitro* + agar	56428	Y	5.88E−01	Positive
*In vitro* + agar	56307C	N	7.44E−05	Positive
*In vitro* + agar	56663C	N	1.14E−05	Negative
*In planta* + sterile	JJ2528	Y	8.78E−07	Positive
*In planta* + sterile	56428	Y	3.38E−04	Negative
*In planta* + sterile	56307C	N	2.61E−08	Negative
*In planta* + sterile	56663C	N	1.01E−07	Positive
*In planta* + nonsterile	JJ2528	Y	1.61E−05	Positive
*In planta* + nonsterile	JJ1886	Y	6.58E−09	Positive
*In planta* + nonsterile	56428	Y	0	Not applicable
*In planta* + nonsterile	56307C	N	0	Not applicable
*In planta* + nonsterile	56663C	N	0	Not applicable

a N, no; Y, yes.

10.1128/mSphere.00329-20.1 FIG S1 Donor strain Ab^zeoR^ and recipient E. coli were cocultured *in vitro* in broth (A) or on agar (B) for horizontal gene transfer. Parent recipient strains without the target plasmid were isolated from singly selective plates. Horizontal gene transformant (with the superscript “zeoR”) isolates were collected from doubly selective plates. Recipient monocultures were also collected from doubly selective plates to verify the spontaneous mutants (SM). Plasmid DNA were extracted, PCR amplified for the zeocin resistance gene, and visualized. Pure pMU125_zeoR served as a positive (+) control. The *Taq* polymerase, primers, and water were run as a negative (-) control. Download FIG S1, TIF file, 2.4 MB. Copyright © 2020 Maeusli et al. 2020 Maeusli et al. This content is distributed under the terms of the Creative Commons Attribution 4.0 International license.

10.1128/mSphere.00329-20.4 TABLE S1 Bacterial strains used in this study. Download Table S1, DOCX file, 0.01 MB. Copyright © 2020 Maeusli et al. 2020 Maeusli et al. This content is distributed under the terms of the Creative Commons Attribution 4.0 International license.

Of the positive PCR confirmed strains, the 2 ESBL-producing and 2 non-ESBL-producing strains with the highest frequencies of dual resistance in broth were selected for *in vitro* agar cocultures. Frequencies of dual antibiotic resistance in agar cocultures were documented (Table 1) for the 3 confirmed transformant strains, JJ2528^zeoR^, 56428^zeoR^, and 56307C^zeoR^ (Fig. S1B). There was no statistically significant difference in the frequencies of dual resistance in liquid-broth versus solid-agar cocultures between the 4 candidate strains (Mann-Whitney test, *P* > 0.05). Thus, the 2 ESBL-producing and 2 non-ESBL-producing strains with the highest frequencies of dual resistance in broth coculture were also selected for *in planta* horizontal gene transfer studies.

### *In planta* transfer of antibiotic resistance genes.

In order to confirm that our model was applicable to the transmission of antibiotic resistance genes on produce, we tested whether horizontal gene transfer could take place on the surface of sterile and nonsterile lettuce leaf discs. Frequencies of dual resistance from coculture on lettuce were generally lower than the results from *in vitro* conditions (Table 1). Strains 56428 and 56307C were confirmed for plasmid uptake *in vitro* but not *in planta*, suggesting that the frequency of plasmid transfer events is negatively affected by the nutrient-depleted leaf surface (Table 1 and Fig. S1). However, 56663C had similar frequencies of dual antibiotic resistance in broth and on sterile lettuce tissue but no plasmid transmission on agar and nonsterile lettuce. To test if the absence of plasmid transfer *in vitro* correlated with an absence of plasmid transfer *in planta*, we tested JJ1886 on nonsterile lettuce as well to model environmental transmission. Surprisingly, JJ1886 demonstrated plasmid uptake on nonsterile lettuce despite demonstrating no *in vitro* broth plasmid transmission (Table 1 and Fig. S2B). JJ2528 demonstrated successful transformation in all *in vitro* and *in planta* conditions (Table 1, Fig. S1A and B, and Fig. S2A and B). Thus, transformant JJ2528^zeoR^ was selected for the *in vivo* model system. No statistically significant difference was found between the *in planta* frequencies of dual resistance of ESBL- and non-ESBL-producing strains on sterile and nonsterile lettuce (Mann-Whitney tests, *P* > 0.05).

10.1128/mSphere.00329-20.2 FIG S2 Donor-recipient combinations were inoculated onto sterile (A) and nonsterile (B) BCL leaf discs, and plasmid transmission events were PCR confirmed. Parent recipient strains without the target plasmid were isolated from singly selective plates. Horizontal gene transformant (with the superscript “zeoR”) CFU isolates were collected from doubly selective plates. Plasmid DNA were extracted, PCR amplified for the zeocin resistance gene, and visualized. Pure pMU125_zeoR served as a positive (+) control. The *Taq* polymerase, primers, and water were run as a negative (-) control. Download FIG S2, TIF file, 1.9 MB. Copyright © 2020 Maeusli et al. 2020 Maeusli et al. This content is distributed under the terms of the Creative Commons Attribution 4.0 International license.

### *In vivo* colonization of mice by transformant E. coli and horizontal gene transfer in the mouse gut microbiome.

**FIG 1:**
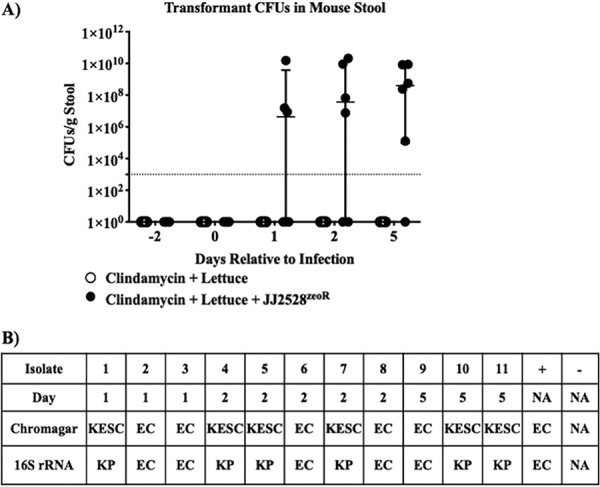
*In vivo* colonization of transformed E. coli and horizontal gene transfer in the mouse gut. (A) Mice (*n* = 12) were treated with 200 μl of 100 mg/kg clindamycin once daily from day −2 to 1. On day 0, mice were orally gavaged with lettuce homogenate with or without 10^7^ CFU E. coli JJ2528^zeoR^. Fecal samples were plated on doubly selective plates for CFU enumeration. The limit of detection was 10^3^ CFU/g stool (dotted line). (B) Eleven CFU isolates were selected for species confirmation via CHROMagar plating and 16S rRNA sequencing. E. coli JJ2528^zeoR^ and E. coli DH5α^ampR^ served as positive (+) and negative (-) controls, respectively. Horizontal gene transmission resulted in antibiotic-resistant K. pneumoniae. Both E. coli and K. pneumoniae persisted through day 5 in the mouse gut. NA, not applicable; KESC, *Klebsiella*, *Enterococcus*, and *Citrobacter*; EC, E. coli; KP, K. pneumoniae.

We then sought to determine whether transformant E. coli from the *in planta* experiment could colonize the mammalian gut. Mice were placed on a 4-day clindamycin regimen to mimic short-course antibiotic treatment in humans. Transformant JJ2528^zeoR^, isolated from the *in planta* experiment, was cultured and homogenized with lettuce tissue. The lettuce homogenate containing E. coli JJ2528^zeoR^ was then fed to mice by oral gavage, and fecal samples were collected on days −2, 0, 1, 2, and 5. Pretreatment and preinfection controls (days −2 and 0) demonstrated no background target antibiotic resistance (Fig. 1A). Days 1, 2, and 5 resulted in 50% (*n* = 3/6), 66.67% (*n* = 4/6), and 83.33% (*n* = 5/6), respectively, of mice being colonized with bacterial strains dually resistant to ampicillin and zeocin. PCR confirmed the presence of pMU125_zeoR in the mouse feces up to 5 days postinfection (Fig. S3). Additionally, *in vivo* transfer of the plasmid to resident Klebsiella pneumoniae, identified by differential culture on CHROMagar medium and confirmed by 16S sequencing, was also observed (Fig. 1B).

10.1128/mSphere.00329-20.3 FIG S3 Eleven CFU isolates were selected from the doubly selective mouse stool plating for plasmid DNA confirmation. Plasmid DNA were extracted, PCR amplified for the zeocin resistance gene, and visualized. E. coli JJ2528^zeoR^ and E. coli DH5α^ampR^ served as positive (+) and negative (-) controls, respectively. Download FIG S3, TIF file, 1.2 MB. Copyright © 2020 Maeusli et al. 2020 Maeusli et al. This content is distributed under the terms of the Creative Commons Attribution 4.0 International license.

We found that the transfer of antibiotic resistance genes can occur between bacterial colonizers of plants and the mammalian gut microbiome via ingestion, during the course of antibiotic treatment. These results highlight the potential contribution of plant foods to the spread of antibiotic resistance. This potential is underscored by the estimated 258.2 million patients prescribed oral antibiotics every year in the United States alone, which could exacerbate the risk of harboring antibiotic resistance genes from bacteria acquired via produce (19).

We showed that the majority of the pathogenic E. coli isolates were able to receive the plasmid from the nonpathogenic donor Ab^zeoR^ in broth (*n* = 8/12) and on agar (*n* = 3/4) *in vitro* cocultures. However, there were considerable differences in the frequencies of dual resistance (Table 1). Furthermore, some strains were better able to receive zeocin resistance under artificial *in vitro* conditions, while other strains did so more effectively on the leaf surface. The lower rates of horizontal gene transfer observed on the lettuce leaves may be due to nutrient-limited stress conditions adversely affecting the fitness and physiology of the recipient bacteria (Table 1 and Fig. S2). Nevertheless, it is possible for antibiotic resistance mobile genes to spread from a nonpathogenic, environmental species to both ESBL- and non-ESBL-producing E. coli on crops. These results underscore the complexity of environmental transfer of antibiotic resistance and highlight the need for more research in this space to determine the drivers of resistance spread under environmental conditions.

While pMU125_zeoR is a laboratory plasmid, we selected it due to the absence of zeocin resistance in the candidate recipient strains and the low frequency of preexisting zeocin resistance in the mouse gut. We have thus established a novel system using Ab^zeoR^ to demonstrate horizontal gene transfer on leaf tissue. An important next step would be to apply this model to naturally occurring plasmids.

In conclusion, while most One Health attention has been on the potential for antibiotic resistance transmission from livestock and contaminated meat products to people, plant foods are fundamental to the food chain for meat eaters and vegetarians alike. Further research into the transmission of antibiotic resistance from plant foods to mammals is needed.

### Bacterial strains and growth conditions.

Bacterial strains used in this study are listed in Table S1. The recipient E. coli strains are all clinical isolates and were randomly selected to include representation by ESBL- and non-ESBL-producing isolates. E. coli strains were grown in tryptic soy broth (TSB; VWR International, 90000-378) at 37°C and 200 rpm or on tryptic soy agar (TSA; VWR International, 90000-786) plates without shaking. A. baylyi was grown in TSB at 30°C and 200 rpm or on TSA plates without shaking unless noted otherwise. One hundred microliters of both the donor and recipient overnight cultures were cocultured in 10 ml of TSB for 2 h at 37°C and 200 rpm. For coculture experiments, the numbers of CFU of donor, recipient, recipient spontaneous mutant, and coculture were determined by culturing on eosin-methylene blue (EMB) (89405-430; VWR International) agar with 25 μg/ml zeocin (ZEO) (R25001; Thermo Fisher Scientific), 100 μg/ml ciprofloxacin (CIP) (AAJ61317-14; Fisher Scientific), or 25 μg/ml ZEO plus 100 μg/ml CIP.

### Construction of the A. baylyi donor strain Ab^zeoR^.

pMU125 is a broad-host-range shuttle vector. pMU125 is a low-copy-number plasmid in A. baylyi and a high-copy-number replicon in E. coli (13, 20). One hundred nanograms of pMU125_zeoR was transformed into 100 μl of A. baylyi ADP1 by electroporation at 1.8 kV, and the bacteria were incubated in Super Optimal broth with catabolite repression medium (SOC) (B9020S; New England BioLabs) for 1 h at 37°C and 200 rpm. Transformants were selected on EMB agar with 100 μg/ml ampicillin (AMP) (A9518; Sigma-Aldrich) plus 50 μg/ml ZEO. Successful transformation of Ab^zeoR^ was verified by plasmid DNA purification and visualization as described below.

### Detection of plasmid transfer.

Recipient monococultures were plated on doubly selective EMB agar plates (100 μg/ml CIP plus 25 μg/ml ZEO) for 48 h at 37°C to account for spontaneous mutants. Coculture CFU that grew on doubly selective plates were indicative of plasmid transfer events. Transfer was confirmed by plasmid purification and PCR. One colony was selected at random per successful coculture condition. Plasmid DNA was harvested from overnight cultures of bacteria, and DNA was purified using the GeneJET plasmid miniprep kit (K0502; Thermo Scientific) per the manufacturer’s protocol. PCR amplification was performed with gene-specific primers targeting the Streptoalloteichus hindustanus *ble* gene (confers zeocin resistance) (Table S2). Plasmid DNA was separated by gel electrophoresis and visualized by SYBR Safe on a gel imager. The frequency of dual antibiotic resistance in coculture was calculated by dividing the transformant density (CFU per milliliter) (coculture with double selection) by the recipient input (CFU per milliliter).

10.1128/mSphere.00329-20.5 TABLE S2 Primers. Download Table S2, DOCX file, 0.01 MB. Copyright © 2020 Maeusli et al. 2020 Maeusli et al. This content is distributed under the terms of the Creative Commons Attribution 4.0 International license.

### *In vitro* coculture experiment.

The *in vitro* coculture experiment was designed as a selection process for candidate recipient strains that would most likely model successful horizontal gene transfer in downstream *in planta* and *in vivo* work. The Ab^zeoR^ donor strain and E. coli recipient strains were grown overnight in TSB as described above (Table 1). Each *in vitro* donor-recipient pair was used in 3 biological replicates. The donor and recipient inocula were quantified by plating serial dilutions in triplicate on selective agar plates as described above. Plasmid transfer was determined as described above using PCR amplification with primers PN0041 and PN0042 (165 bp).

Recipient strains JJ2528, 56428, 56663C, and 56307C were selected for horizontal gene transfer coculture on agar. Donor-recipient cocultures were grown overnight on nonselective TSA plates using a modified published protocol (21). Horizontal gene transfer was confirmed by scraping, resuspending, and plating the coculture on doubly selective EMB agar plates as previously described. Plating and plasmid transfer confirmations were performed using the same methods as for *in vitro* broth cocultures.

### *In planta* coculture experiment.

Commercial lettuce contains high numbers of colonizing bacteria (22), which would have confounded our analysis of plasmid transfer. We therefore grew a commercial variety of Lactuca sativa, commonly known as Buttercrunch lettuce (BCL), from seed in the lab as previously described (23). In short, plants were cultured in a plant growth chamber (MLR-352-PA; Panasonic), and conditions were adjusted to a 16-h photoperiod, 16 to 24°C, and 60 to 85% relative humidity (RH), to mimic field conditions (24).

Leaf discs were collected using a cap punch method in a 2.0-ml microcentrifuge tube, and then half of the discs were surface sterilized in 70% ethanol for 30 s to eliminate background leaf microbiota. The sterilized leaf discs were air dried, and 20 μl of either phosphate-buffered saline (PBS) (negative control), Ab^zeoR^ (donor), E. coli (recipient), or a 1:1 coculture of donor and recipient bacteria was drop-inoculated onto the surfaces of leaf discs and incubated overnight at 30°C. Each inoculum condition was used in duplicate. All bacterial cultures were normalized by absorbance (optical density at 600 nm [OD_600_] = 0.5) prior to inoculation onto the lettuce leaf discs. Bacterial inocula were verified by performing serial dilutions in PBS and plating in triplicate on TSA plates. Plates were incubated overnight at 30°C and 37°C for Ab^zeoR^ and recipient E. coli, respectively, and CFU were enumerated. Last, leaf discs were homogenized in 1 ml PBS, and serial dilutions of the lettuce homogenate were plated in triplicate on singly and doubly selective EMB agar plates as described above. Plasmid transfer was confirmed as described above using PCR amplification with primers PN0041 and PN0042 (165 bp).

### *In vivo* colonization of mutant E. coli in mice.

Female, murine-pathogen-free BALB/c mice, 8 to 10 weeks of age, were obtained from Taconic Biosciences. A total of 12 mice (6 mice per treatment group) were used for the *in vivo* study. Adapted from previous publications, mice were treated with 200 μl of 100 mg/kg clindamycin once daily via subcutaneous injection for 4 days starting 2 days before infection and continuing through 1 day postinfection (25–27). Sterilized leaf tissue was homogenized in PBS to a concentration of 0.33 g/ml, which was the maximum density that could be used without compromising proper function of the feeding needles. E. coli JJ2528^zeoR^ (isolated from the *in planta* assay) was mixed with the lettuce homogenate at the time of oral gavage to mimic consumption of produce contaminated with antibiotic-resistant bacteria. Mice were orally gavaged with 100 μl lettuce homogenate with or without 10^7^ CFU JJ2528^zeoR^.

One stool pellet per mouse was collected on days −2, 0, 1, 2, and 5 for CFU quantification. Additionally, mouse stool pellets were collected before oral gavage on day 0. All fecal samples collected on day 0 were collected immediately before infection. Fecal samples were collected between 7 and 10 a.m., as described by others (28). Fecal pellets were homogenized in PBS by vortexing at maximum speed for 5 min. Serial dilutions were performed and plated in triplicate on EMB agar with 100 μg/ml AMP plus 50 μg/ml ZEO, and the plates were incubated overnight at 37°C.

Eleven isolates were selected at random from the doubly selective plates from days 1, 2, and 5, and horizontal gene transfer was confirmed by plasmid purification and PCR. Plasmid DNA were extracted, PCR amplified for the zeocin resistance gene with primers LEE35 and LEE36 (250 bp), and visualized on an ethidium bromide gel. E. coli JJ2528^zeoR^ and E. coli DH5α^ampR^ served as positive and negative controls, respectively. These isolates were subcultured on chromogenic differential plates (Orientation; CHROMagar), and species identification was confirmed by Sanger sequencing of the 16S rRNA region (Genewiz).

## References

[1] Spellberg B, Hansen GR, Kar A, Cordova CD, Price LB, Johnson JR 2016 Antibiotic resistance in humans and animals. NAM Perspectives doi:10.31478/201606d.

[2] Centers for Disease Control and Prevention. 2013 Antibiotic resistance from the farm to the table. Centers for Disease Control and Prevention, Atlanta, GA.

[3] Centers for Disease Control and Prevention. 2013 Antibiotic resistance threats in the United States. Centers for Disease Control and Prevention, Atlanta, GA.

[4] U.S. Food and Drug Administration. 2016 2015 summary report on antimicrobials sold or distributed for use in food-producing animals. Food and Drug Administration, Washington, DC.

[5] Laxminarayan R, Duse A, Wattal C, Zaidi AKM, Wertheim HFL, Sumpradit N, Vlieghe E, Hara GL, Gould IM, Goossens H, Greko C, So AD, Bigdeli M, Tomson G, Woodhouse W, Ombaka E, Peralta AQ, Qamar FN, Mir F, Kariuki S, Bhutta ZA, Coates A, Bergstrom R, Wright GD, Brown ED, Cars O 2013 Antibiotic resistance-the need for global solutions. Lancet Infect Dis 13:1057–1098. doi:10.1016/S1473-3099(13)70318-9. 24252483

[6] Hölzel CS, Tetens JL, Schwaiger K 2018 Unraveling the role of vegetables in spreading antimicrobial-resistant bacteria: a need for quantitative risk assessment. Foodborne Pathog Dis 15:671–688. doi:10.1089/fpd.2018.2501. 30444697 PMC6247988

[7] Liu CM, Stegger M, Aziz M, Johnson TJ, Waits K, Nordstrom L, Gauld L, Weaver B, Rolland D, Statham S, Horwinski J, Sariya S, Davis GS, Sokurenko E, Keim P, Johnson JR, Price LB 2018 *Escherichia coli* ST131-H22 as a foodborne uropathogen. mBio 9:e00470-19. doi:10.1128/mBio.00470-18. 30154256 PMC6113624

[8] O’Neill J 2015 Antimicrobials in agriculture and the environment: reducing unnecessary use and waste. The Review on Antimicrobial Resistance, United Kingdom.

[9] Centers for Disease Control and Prevention. 2019 Antibiotic resistance threats in the United States. Centers for Disease Control and Prevention, Atlanta, GA.

[10] Carvalheira A, Silva J, Teixeira P 2017 Lettuce and fruits as a source of multidrug resistant *Acinetobacter* spp. Food Microbiol 64:119–125. doi:10.1016/j.fm.2016.12.005. 28213015

[11] Gu G, Ottesen A, Bolten S, Ramachandran P, Reed E, Rideout S, Luo Y, Patel J, Brown E, Nou X 2018 Shifts in spinach microbial communities after chlorine washing and storage at compliant and abusive temperatures. Food Microbiol 73:73–84. doi:10.1016/j.fm.2018.01.002. 29526229

[12] Cooper RM, Tsimring L, Hasty J 2017 Inter-species population dynamics enhance microbial horizontal gene transfer and spread of antibiotic resistance. Elife 6:e25950. doi:10.7554/eLife.25950. 29091031 PMC5701796

[13] Fulsundar S, Harms K, Flaten GE, Johnsen PJ, Chopade BA, Nielsen KM 2014 Gene transfer potential of outer membrane vesicles of *Acinetobacter baylyi* and effects of stress on vesiculation. Appl Environ Microbiol 80:3469–3483. doi:10.1128/AEM.04248-13. 24657872 PMC4018862

[14] Tran F, Boedicker JQ 2019 Plasmid characteristics modulate the propensity of gene exchange in bacterial vesicles. J Bacteriol 201:e00430-18. doi:10.1128/JB.00430-18. PMC6416910 30670543

[15] Dijkshoorn L, Nemec A, Seifert H 2007 An increasing threat in hospitals: multidrug-resistant *Acinetobacter baumannii*. Nat Rev Microbiol 5:939–951. doi:10.1038/nrmicro1789. 18007677

[16] Forsberg KJ, Reyes A, Wang B, Selleck EM, Sommer MOA, Dantas G 2012 The shared antibiotic resistome of soil bacteria and human pathogens. Science 337:1107–1111. doi:10.1126/science.1220761. 22936781 PMC4070369

[17] Partridge SR, Kwong SM, Firth N, Jensen SO 2018 Mobile genetic elements associated with antimicrobial resistance. Clin Microbiol Rev 31:e00088-17. doi:10.1128/CMR.00088-17. 30068738 PMC6148190

[18] Manyi-Loh C, Mamphweli S, Meyer E, Okoh A 2018 Antibiotic use in agriculture and its consequential resistance in environmental sources: potential public health implications. Molecules 23:795. doi:10.3390/molecules23040795. PMC6017557 29601469

[19] Centers for Disease Control and Prevention. 2019 Outpatient antibiotic prescriptions. Centers for Disease Control and Prevention, Atlanta, GA.

[20] Dorsey CW, Tomaras AP, Actis LA 2002 Genetic and phenotypic analysis of *Acinetobacter baumannii* insertion derivatives generated with a transposome system. AEM 68:6353–6360. doi:10.1128/AEM.68.12.6353-6360.2002. PMC134429 12450860

[21] Leonard S, Barrick J, Perreau J, Elston K, Corley K 2020 General conjugation protocol. Barrick lab https://barricklab.org/twiki/bin/view/Lab/ProtocolsConjugation.

[22] Uhlig E, Olsson C, He J, Stark T, Sadowska Z, Molin G, Ahrné S, Alsanius B, Håkansson Å 2017 Effects of household washing on bacterial load and removal of *Escherichia coli* from lettuce and “ready-to-eat” salads. Food Sci Nutr 5:1215–1220. doi:10.1002/fsn3.514. 29188050 PMC5694878

[23] Dimopoulou A, Theologidis I, Liebmann B, Kalantidis K, Vassilakos N, Skandalis N 2019 *Bacillus amyloliquefaciens* MBI600 differentially induces tomato defense signaling pathways depending on plant part and dose of application. Sci Rep 9:19120. doi:10.1038/s41598-019-55645-2. 31836790 PMC6910970

[24] Beris D, Theologidis I, Skandalis N, Vassilakos N 2018 *Bacillus amyloliquefaciens* strain MBI600 induces salicylic acid dependent resistance in tomato plants against Tomato spotted wilt virus and Potato virus Y. Sci Rep 8:10320. doi:10.1038/s41598-018-28677-3. 29985434 PMC6037670

[25] Hertz FB, Løbner-Olesen A, Frimodt-Møller N 2014 Antibiotic selection of *Escherichia coli* sequence type 131 in a mouse intestinal colonization model. Antimicrob Agents Chemother 58:6139–6144. doi:10.1128/AAC.03021-14. 25092712 PMC4187947

[26] Hoyen CK, Pultz NJ, Paterson DL, Aron DC, Donskey CJ 2003 Effect of parenteral antibiotic administration on establishment of intestinal colonization in mice by *Klebsiella pneumoniae* strains producing extended-spectrum beta-lactamases. Antimicrob Agents Chemother 47:3610–3612. doi:10.1128/aac.47.11.3610-3612.2003. 14576127 PMC253805

[27] Pultz MJ, Nerandzic MM, Stiefel U, Donskey CJ 2008 Emergence and acquisition of fluoroquinolone-resistant gram-negative bacilli in the intestinal tracts of mice treated with fluoroquinolone antimicrobial agents. Antimicrob Agents Chemother 52:3457–3460. doi:10.1128/AAC.00117-08. 18606843 PMC2533488

[28] Hart ML, Meyer A, Johnson PJ, Ericsson AC 2015 Comparative evaluation of DNA extraction methods from feces of multiple host species for downstream next-generation sequencing. PLoS One 10:e0143334. doi:10.1371/journal.pone.0143334. 26599606 PMC4657925

