# Supplementary figures and images for: Horizontal Gene Transfer of Antibiotic Resistance from Acinetobacter baylyi to Escherichia coli on Lettuce and Subsequent Antibiotic Resistance Transmission to the Gut Microbiome
Source: mSphere. 2020 May 27;5(3):e00329-20. doi: 10.1128/mSphere.00329-20 (PMC7253597; doi:10.1128/mSphere.00329-20)

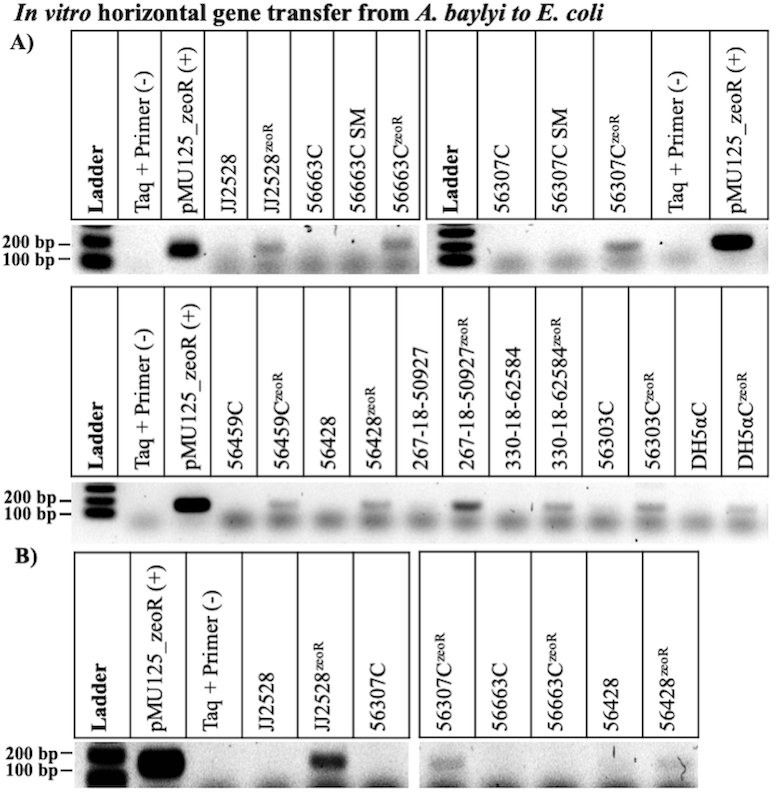

Supplement: FIG S1 [file mSphere.00329-20-sf001.tif]

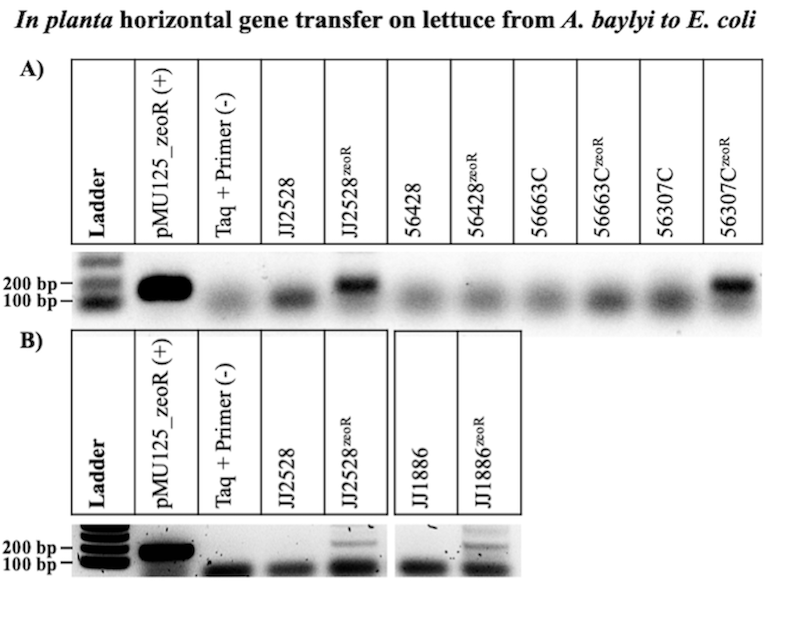

Supplement: FIG S2 [file mSphere.00329-20-sf002.tif]

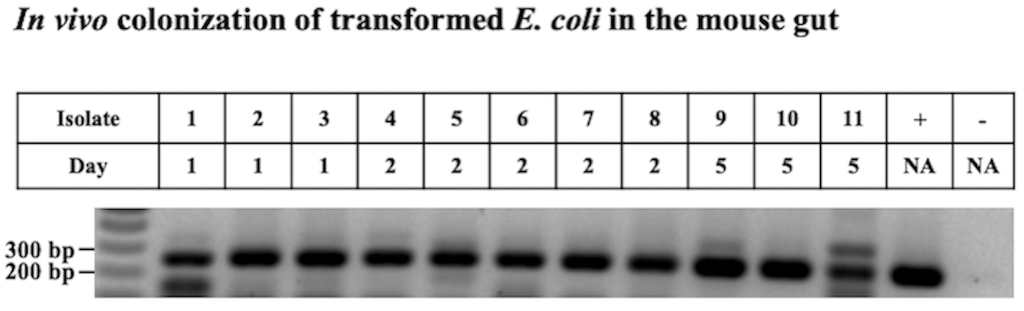

Supplement: FIG S3 [file mSphere.00329-20-sf003.tif]
