# Supplementary material for: Horizontal Gene Transfer of Antibiotic Resistance from Acinetobacter baylyi to Escherichia coli on Lettuce and Subsequent Antibiotic Resistance Transmission to the Gut Microbiome
Source: mSphere. 2020 May 27;5(3):e00329-20. doi: 10.1128/mSphere.00329-20 (PMC7253597; doi:10.1128/mSphere.00329-20)
Supplement: TABLE S1 [file mSphere.00329-20-st001.docx]

| **Table S1: Summary of bacterial strains used in this study** | | |
| --- | --- | --- |
| **Strains** | **Description** | **Reference** |
| *A. baylyi* ADP1 | Environmental bacteria | ATCC 33305 |
| Ab^zeoR^ | *A. baylyi* ADP1 transformed with pMU125_zeoR | This study |
| *E. coli* DH5α | Competent strain used for transformation | Thermo Fisher |
| *E. coli* DH5α^ampR^ | *E. coli* DH5α transformed with pMU125 | REF(1) |
| *E. coli* DH5αC | CIP resistant mutant DH5α generated by serial passage | This study |
| *E. coli* JJ2528 | ESBL clinical isolate | Gift from J. Johnson |
| *E. coli* JJ2528^zeoR^ | Isolated from *in planta* lettuce experiment. Harbors pMU125_zeoR. | This study |
| *E. coli* JJ1886 | Blood and urine clinical isolate. ESBL. ST131, CTX-M-15-producing H30-Rx sublineage. | REF(2) |
| *E. coli* JJ2555 | ESBL clinical isolate | Gift from J. Johnson |
| *E. coli* 267-18-50927 | ESBL clinical isolate | Gift from R. She |
| *E. coli* 330-18-62584 | ESBL clinical isolate | Gift from R. She |
| *E. coli* 56307 | Non-ESBL clinical isolate | Gift from R. She |
| *E. coli* 56307C | CIP resistant mutant 56307 generated by serial passage | This study |
| *E. coli* 56459 | Non-ESBL clinical isolate | Gift from R. She |
| *E. coli* 56459C | CIP resistant mutant 56459 generated by serial passage | This study |
| *E. coli* 56303 | Non-ESBL clinical isolate | Gift from R. She |
| *E. coli* 56303C | CIP resistant mutant 56303 generated by serial passage | This study |
| *E. coli* 56663 | Non-ESBL clinical isolate | Gift from R. She |
| *E. coli* 56663C | CIP resistant mutant 56663 generated by serial passage | This study |
| *E. coli* 267-19-46076 | ESBL clinical isolate | Gift from R. She |
| *E. coli* 267-19-44723 | ESBL clinical isolate | Gift from R. She |
| *E. coli* 56428 | ESBL clinical isolate | Gift from R. She |
| **Plasmids** | **Description** | **Reference** |
| pMU125 | AMP resistance | REF(1) |
| pMU125_zeoR | Encodes gfp reporter. AMP and ZEO resistance. | REF(3) |

**References**

1. [Dorsey CW, Tomaras AP, Actis LA. 2002. Genetic and phenotypic analysis of *Acinetobacter baumannii* insertion derivatives generated with a transposome system. Appl Environ Microbiol 68:6353–6360.](http://paperpile.com/b/5bGeFQ/VjqDQ)

2. [Owens RC Jr, Johnson JR, Stogsdill P, Yarmus L, Lolans K, Quinn J. 2011. Community transmission in the United States of a CTX-M-15-producing sequence type ST131 *Escherichia coli* strain resulting in death. J Clin Microbiol 49:3406–3408.](http://paperpile.com/b/C28lnQ/ooSM)

3. [Luna BM, Ulhaq A, Yan J, Pantapalangkoor P, Nielsen TB, Davies BW, Actis LA, Spellberg B. 2017. Selectable Markers for Use in Genetic Manipulation of Extensively Drug-Resistant (XDR) *Acinetobacter baumannii* HUMC1. mSphere 2.](http://paperpile.com/b/C28lnQ/eke1)
