# Supplementary material for: Horizontal Gene Transfer of Antibiotic Resistance from Acinetobacter baylyi to Escherichia coli on Lettuce and Subsequent Antibiotic Resistance Transmission to the Gut Microbiome
Source: mSphere. 2020 May 27;5(3):e00329-20. doi: 10.1128/mSphere.00329-20 (PMC7253597; doi:10.1128/mSphere.00329-20)
Supplement: TABLE S2 [file mSphere.00329-20-st002.docx]

| **Table S2: Primers** | | | | | |
| --- | --- | --- | --- | --- | --- |
| **Primer Name** | **Plasmid** | **Target** | **Primer Direction** | **Sequence** | **Source** |
| LEE35 | pMU125_zeoR | ZEO resistance cassette | Forward | CAAGTTGACCAGTGCCGTTC | This study |
| LEE36 | pMU125_zeoR | ZEO resistance cassette | Reverse | GTTCGTGGACACGACCTCC | This study |
| PN0041 | pMU125_zeoR | ZEO resistance cassette | Forward | CGACGTGACCCTGTTCATC | REF(1) |
| PN0042 | pMU125_zeoR | ZEO resistance cassette | Reverse | TCGCCGATCTCGGTCAT | REF(1) |

**References**

1. [Luna BM, Ulhaq A, Yan J, Pantapalangkoor P, Nielsen TB, Davies BW, Actis LA, Spellberg B. 2017. Selectable Markers for Use in Genetic Manipulation of Extensively Drug-Resistant (XDR) *Acinetobacter baumannii* HUMC1. mSphere 2.](http://paperpile.com/b/C28lnQ/eke1)
